# Association between NF-κB Pathway Gene Variants and sICAM1 Levels in Taiwanese

**DOI:** 10.1371/journal.pone.0169516

**Published:** 2017-01-17

**Authors:** Semon Wu, Ming-Sheng Teng, Leay-Kiaw Er, Wan-Yi Hsiao, Lung-An Hsu, Ching-Hua Yeh, Jeng-Feng Lin, Yi-Ying Lin, Cheng-Wen Su, Yu-Lin Ko

**Affiliations:** 1 Department of Life Science, Chinese Culture University, Taipei, Taiwan; 2 Department of Research, Taipei Tzu Chi Hospital, Buddhist Tzu Chi Medical Foundation, New Taipei city, Taiwan; 3 The Division of Endocrinology and Metabolism, Department of Internal Medicine, Taipei Tzu Chi Hospital, Buddhist Tzu Chi Medical Foundation, New Taipei City, Taiwan; 4 The First Cardiovascular Division, Department of Internal Medicine, Chang Gung Memorial Hospital and Chang Gung University College of Medicine, Taoyuan, Taiwan; 5 Cardiovascular Center and Division of Cardiology, Department of Internal Medicine and, Taipei Tzu Chi Hospital, Buddhist Tzu Chi Medical Foundation, New Taipei city, Taiwan; 6 School of Medicine, Tzu Chi University, Hualien, Taiwan; Nagoya University, JAPAN

## Abstract

Intercellular adhesion molecule–1 (ICAM1) is crucial to the development and progression of atherosclerosis. Recent genome-wide association studies (GWAS) have revealed that single nucleotide polymorphisms (SNPs) in two of the nuclear factor-κB (NF-κB) pathway genes, *NFKBIK* and *RELA*, are associated with soluble ICAM1 (sICAM1) levels. However, neither of these two gene variants is found in the Asian populations. This study aimed to elucidate whether other candidate gene variants involved in the NF-κB pathway may be associated with sICAM1 levels in Taiwanese. After excluding carriers of the *ICAM1* rs5491-T allele, three SNPs in the *ICAM1* gene and eight SNPs in six of the NF-κB pathway genes (*NFKB1*, *PDCD11*, *TNFAIP3*, *NKAPL*, *IKBKE*, and *PRKCB*) were analyzed for their association with sICAM1 levels in 480 individuals. Our data showed that two SNPs, rs5498 of *ICAM1* and rs1635 of *NKAPL*, were significantly associated with sICAM1 levels (*P* = 0.002 and 0.004, respectively) in the Taiwanese population. Using a multivariate analysis, rs5498 and rs1635 as well as the previously reported *ABO* genotypes and rs12051272 of the *CDH13* gene were independently associated with sICAM1 levels (*P* = 0.001, 0.001, 0.006 and 0.031, respectively). An analysis with combined risk alleles of four candidate SNPs in the *ICAM1*, *NKAPL*, *ABO*, and *CDH13* genes showed an increase in sICAM1 levels with added numbers of risk alleles and weighted genetic risk score. Our findings thus expanded the repertoire of gene variants responsible for the regulation of sICAM1 levels in the Asian populations.

## Introduction

ICAM1 is an adhesive molecule of the immunoglobulin superfamily that is regulated by the proinflammatory cytokines and plays a crucial role in the development and progression of atherosclerosis [1]. Apart from atherosclerosis, elevated plasma sICAM1 levels were observed in patients with unstable angina and myocardial infarction, and they served as indications for an increased risk of ischemic stroke, myocardial infarction, and future coronary events in both healthy individuals and patients with coronary heart disease [2–5]. A study of the Framingham cohort has shown a cumulative effect on plasma sICAM1 levels exerted by quantitative combination of a number of clinical risk factors, suggestive of a multitude of physiological control over ICAM1 protein expression and its post-translational processing [6].

In addition to physiological regulations, a 24% residual heritability of sICAM1was found in the Framingham cohort [6], which provided one of the earliest evidence of the genetic regulation of sICAM1 concentration. Several studies subsequently narrowed down the genetic determinants of circulating sICAM1 levels to the *ICAM* cluster on chromosome 19 with a heritability estimate from 0.34 to 0.59 [7, 8], and many single nucleotide polymorphisms (SNPs) in the vicinity of the *ICAM1* locus were identified to associate with sICAM1 levels [7, 9–11]. We have previously reported that the *ICAM1* SNP rs5491 was associated with sICAM1 levels and metabolic syndrome in a Taiwanese population [12]. This SNP, also named ICAM1^Kilifi^ (rs5491), lies in a loop close to the N-terminus of the ICAM1 protein critical to its binding to the malaria parasite *Plasmodium falciparum*, human rhinovirus, lymphocyte function–associated antigen–1 (LFA-1), and fibrinogen [13–15], and is likely a result of evolutionary adaptation. Unfortunately, the rs5491-T allele creates a non-synonymous lysine-to-methionine substitution that was known to interfere with binding of an ICAM1 epitope to several monoclonal antibodies used in conventional protein assays [16]. Therefore, the presence of this allele might confound interpretation of our previous results.

Numerous efforts have been made toward identifying novel genetic regulators for sICAM1 levels. For instance, variants of the *ABO* and *CDH13* genes (which encode the ABO histo-blood group antigen and glycosylphosphatidylinositol-anchored cadherin 13, respectively) were reported to associate with sICAM1 levels [10, 17, 18]. Recently, a GWAS identified and localized the genetic determinants of sICAM1 levels to several loci in the genome, including two SNPs, rs3136642 and rs1049728, in the *NFKBIK* and *RELA* genes which function in the NF-κB pathway [11]. This is interesting because in the cardiovascular system, ICAM1 expression in the endothelial cells is known to be upregulated by various extracellular signals like tumor necrosis factor-alpha and thrombin, and the majority of these signals lead to activation of NF-κB [19]. Noticeably, the promoter region of *ICAM1* contains two NF-κB binding sites, which are -533 and -223 bases from translational start site. It has been shown by site-directed mutagenesis and gel shift assays that RelA/p65 binds to the downstream site to activate *ICAM1* transcription [20]. Since sICAM1 expressed by endothelial cells greatly facilitates transendothelial migration of leukocytes [19], studying the relation between NF-κB pathway activation and ICAM1expression thus allows us to identify key players in this inflammatory process. However, neither of these SNPs (rs3136642 and rs1049728) is present in the Asian populations.

The NF-κB family transcription factors are central regulators of a variety of genes which are essential to processes like inflammation, immunity, cell proliferation, differentiation, and survival [21]. The NF-κB transcription factors are distinct homo- or heterodimers formed by the combination of RelA (p65; a product of the *RELA* gene), RelB, c-Rel, NF-κB1 (p105/p50; encoded by the *NFKB1* gene), and NF-κB2 (p100/p52) proteins. At its simplest, the NF-κB dimer is normally localized in the cytoplasm, where it is kept inactive by the IκB family of inhibitors. Upon signal transduction following extracellular stimuli, IκB is quickly phosphorylated by the IκB kinase (IKK) complexes and degraded, and the NF-κB dimer translocates into the nucleus to function as a transcriptional activator or repressor for downstream genes [21].

On top of these core members of the NF-κB pathway, additional proteins participate in different steps of the NF-κB signal transduction, and many SNPs of these NF-κB pathway genes have been reported to associate with inflammation-related autoimmune diseases. For example, Protein Kinase C-β, encoded by the *PRKCB* gene, recruits IKK into lipid rafts and is involved in B-cell receptor (BCR)-mediated NF-κB activation, and the SNP rs16972959 was shown to associate with systemic lupus erythematosus (SLE) in a Han Chinese population [22]. Polymorphisms in the tumor necrosis factor-alpha-induced protein 3 (*TNFAIP3*), a negative regulator of NF-κB activation, were also found to associate with SLE [23]. Moreover, suggestive associations were found between a SNP of the *NFKB1* gene and primary Sjögren’s syndrome [24], and between SNPs of the *IKBKE* gene (encoding the inhibitor of kappa light polypeptide gene enhancer in B-cells, kinase epsilon; a noncanonical IKK) and rheumatoid arthritis [25]. It remains unclear if any of these SNPs is associated with sICAM1 levels in the Taiwanese population.

In order to further characterize the genetic determinants of sICAM1 levels in Taiwanese, we excluded carriers of the rs5491-T allele and tested the relationship between a panel of eleven SNPs in *ICAM1* and selected NF-κB pathway genes and circulating sICAM1 levels in 480 individuals.

## Materials and Methods

### Subjects

All of the participants responded to a questionnaire on their medical history and lifestyle characteristics, and were recruited during routine health examinations between October 2003 and September 2005 at the Chang Gung Memorial Hospital. They underwent physical examinations in which height, weight, waist and hip circumferences, and blood pressure (BP) in the sitting position after 15 min of rest were measured. Fasting blood samples were obtained from each subject. Exclusion criteria included a history of myocardial infarction, stroke or transient ischemic attack, cancer, and current renal or liver disease. After 617 individuals were initially recruited, we additionally excluded those who took regular medications for diabetes mellitus, hypertension, and lipid-lowering drugs (8, 6, and 60 individuals, respectively), aged < 18 years (5 individuals), and carried the *ICAM1* rs5491-T alleles (58 individuals). At the end, 480 Taiwanese subjects of Han Chinese origin (242 men, mean age: 43.6 ± 9.5 years; 238 women, mean age: 45.9 ± 9.5 years) were enrolled for analyses. Obesity was defined as a body mass index (BMI) ≥ 25 kg/m^2^ according to the Asian criteria [26]. Current smokers were defined as those who smoked regularly at the time of survey. All subjects provided written informed consent. The study was approved by the Ethics Committee of the Tzu-Chi Memorial Hospital (IRB number: 04-XD01-001). The clinical characteristics and biometrics of the study population are summarized in Table 1.

**Table 1:** Clinical and biochemical characteristics of the study population.

	Total	Men	Women	*P* value
Number	480	242	238	
Age (years)	44.74 ±9.55	43.63 ±9.51	45.87 ±9.48	0.01
BMI (kg/m^2^)	24.14 ±3.52	24.90 ±3.24	23.38 ±3.64	1.8×10^−6^
Current smokers (%)	20	35.5	4.2	3.56×10^−15^
Hypertension (%)	9.6	9.1	10.1	0.758
Diabetes mellitus (%)	2.7	2.9	2.5	1
CRP (mg/L)	1.62 ±6.68	1.95 ±8.87	1.29 ±3.15	0.05
Fibrinogen, μmol/L	261.94 ± 67.82	258.14 ± 69.23	265.80 ±66.28	0.216
sE-selectin(μg/L)	52.45 ±24.87	59.51 ± 25.63	45.25 ± 21.89	3.07×10^−12^
sP-selectin(ng/mL)	135.24 ±112.37	146.94 ± 123.50	123.35 ± 98.63	0.007
SAA (μmol/L)	5.82 ±15.56	6.49 ± 19.79	5.16 ± 9.68	0.573
sICAM1 (μg/L)	248.52 ±111.83	254.17 ± 106.20	242.80 ± 117.21	0.090
sVCAM1(μg/L)	486.77 ±134.46	487.21 ± 155.36	486.33 ± 109.64	0.833
MMP-1 (pg/mL)	502.80 ±1256.68	362.48 ± 611.92	645.48 ± 1664.47	0.988
MMP-2 (ng/mL)	126.56 ±41.60	122.74±42.80	130.42 ± 40.08	0.009
MMP-9 (mg/L)	144.73 ±115.15	156.73±116.91	132.63 ±112.29	0.023
MCP-1 (pg/mL)	73.96 ± 62.14	81.77±73.16	66.02 ±47.28	0.002
sTNFRII (pg/mL)	3230.62 ±923.52	3327.01 ± 989.43	3132.60 ± 842.10	0.024

BMI, body mass index; CRP, C-reactive protein; SAA, serum amyloid A; sE-selectin, soluble E-selectin; sP-selectin, soluble P-selectin; sICAM1, soluble intercellular adhesive molecule 1; sVCAM1, soluble vascular cell adhesive molecule 1; MMP1, matrix metalloproteinase 1; MMP2, matrix metalloproteinase 2; MMP9, matrix metalloproteinase 9; MCP-1, Monocyte chemotactic protein-1; sTNFRII, soluble tumor necrosis factor-alpha receptor 2. Continuous variables are presented as mean ± SD. CRP, SAA, sICAM1, sVCAM1,sE-selectin, sP-selectin, MMP1, MMP2, MMP9, MCP1, and sTNFRII values were logarithmically transformed before statistical testing to meet the assumption of normal distributions; however, the untransformed data are shown.

### Genomic DNA extraction and genotyping

Genomic DNA was extracted as previously reported. By candidate gene approaches, in addition to the *ICAM1* gene, NF-κB pathway genes that have been previously found to be associated with inflammation-related autoimmune diseases in genetic associated studies were also selected for analysis. Oligonucleotides were generated to amplify genomic DNA fragments encompassing the SNPs of interest according to the NCBI SNP database (http://www.ncbi.nlm.nih.gov/SNP). In addition to *ICAM1* rs5491, ten SNPs in the *ICAM1* and the NF-κB pathway genes associated with sICAM1’s transcript and protein levels were selected for genotyping (Table 2). Four SNPs in the vicinity of the *ICAM1* loci were selected based on the following criteria: (1) the most significant SNPs associated with ICAM1 levels in previous reports [7, 27]; (2) compatibility with TaqMan assays; (3) cis-expression quantitative trait loci (cis-eQTL) effect in whole blood cells in the GTEx database (http://www.gtexportal.org); (4) a threshold of minor allele frequency (MAF) ≥ 0.10 according to the Chinese (CHB) genotype data from HapMap (http://www.hapmap.org/). Six variants in various NF-κB pathway genes were selected based on the following criteria: (1) functional analyses and genome-wide association study (GWAS) data associated with rheumatic, immune, or psychiatric disorders; (2) with the exception of rs28720239, compatibility with TaqMan assays; (3) a threshold of MAF ≥ 0.05 according to the CHB genotype data from HapMap. Genotyping of rs28720239 was performed using polymerase chain reactions (PCR) with restriction enzyme digestion. Genotyping of rs2271751, rs7894407, rs7089271, rs2230926, rs1635, rs12142086, rs5496, rs5498, rs281432, and rs16972959 was performed using TaqMan SNP Genotyping Assays from Applied Biosystem (ABI, Foster City, CA, USA). Genotyping data are available in S1 Table.

**Table 2:** Association between SNPs in the *ICAM1* and NF-κB pathway genes and sICAM1 levels in the study subpopulation without the *ICAM1* rs5491-T carriers.

SNP	Gene	Position	Major allele	Minor allele(s)	MAF	β(SE)	*P* value ^a^	Adjusted *P* value	Reference
rs5496	*ICAM1*	intron	A	G	0	-		**-**	
rs5498	*ICAM1*	exon 6	A	G	0.246	0.042(0.013)	0.002	0.020	[9, 10]
rs281432	*ICAM1*	intron	C	G	0.286	0.024(0.012)	0.052	0.520	[9, 10]
rs281438	*ICAM4*	3’-UTR	T	G	0.129	0.024(0.016)	0.150	1	GTEx database
rs28720239	*NFkB1*	promoter	-	ATTG	0.424	0.008(0.011)	0.501	1	[11]
rs2271751	*PDCD11*	intron	T	C	0.349	0.003(0.011)	0.814	1	[53]
rs2230926	*TNFAIP3*	exon 4	T	G/C	0.04^b^	0.030(0.027)	0.268	1	[21]
rs1635	*NKAPL*	exon 1	G	T	0.364	-0.031(0.011)	0.004	0.040	[28]
rs12142086	*IKBKE*	intron	C	T	0.316	0.007(0.012)	0.535	1	[23]
rs16972959	*PRKCB*	intron	G	A	0.257	-0.014(0.012)	0.231	1	[20]

MAF: minor allele frequency;

^a:^ adjusted for sex, age, BMI, and smoking status;

^b:^ MAF of the rs2230926-G allele.

Adjusted P value were shown with Bonferroni corrections.

### Laboratory examinations and assays

Medical history and blood samples were obtained as previously reported [28]. The levels of most markers, including serum C-reactive protein (CRP), serum amyloid A (SAA), sICAM1, soluble vascular cell adhesive molecule (sVCAM1), soluble E-selectin (sE-selectin), matrix metalloproteinase 2 (MMP-2), and matrix metalloproteinase 9 (MMP-9), were measured using a sandwich enzyme-linked immunosorbent assay (ELISA) developed in-house (S1 Materials and Methods). The levels of circulating plasma matrix metalloproteinase 1 (MMP-1), monocyte chemotactic protein 1 (MCP-1), soluble P-selectin (sP-selectin), and soluble tumor necrosis factor receptor II (sTNFRII) were measured using commercially available ELISA kits from R&D (Minneapolis, MN, USA). The mean intra-assay CVs from serum specimens were 7.1, 8.5, 2.1, 4.2, 4.1, 6.1, and 7.1%, and the mean inter-assay CVs were 9.5, 8.1, 3.8, 6.8, 3.4, 8.8, and 9.1% for CRP, SAA, sICAM1, sVCAM1, sE-selectin, MMP-2, and MMP-9 levels, respectively. The mean intra-assay CVs from plasma specimens were 3.8, 3.0, 2.2, 3.1, and 5.5%, and the mean inter-assay CVs were 5.7, 8.8, 4.1, 4.5, and 8.2% for MCP-1, sP-selectin, sTNFRII, and MMP-1 levels, respectively.

### Statistical analyses

The chi-square test was used to compare categorical variables of smoking status, hypertension, and diabetes mellitus. The clinical characteristics of the continuous variables were expressed as means ± SD and tested using two-sample t-test or analysis of variance (ANOVA). Pearson and partial correlation coefficients were used to examine the relationship between sICAM1 levels and clinical and biochemical factors. A generalized linear model was used to analyze levels of sICAM1 in relation to predictors of the investigated genotypes and confounders. Additive models were used for numeric association tests after recoding our SNPs from categorical variables to continuous variables, such as 0, 1 and 2 of a particular allele. The Bonferroni method was used to correct for multiple comparisons where applicable. The genetic risk scores were created using two methods: a simple count method (genetic risk allele: GRA) and a weighted method (genetic risk score: GRS) [29, 30]. Both methods assumed each SNP to be independently associated with sICAM1 levels (i.e., no interaction between SNPs). We assumed an additive effect of risk alleles for each SNP, and applied linear weighting of 0, 1, or 2 to genotypes containing corresponding number of risk alleles. The count model assumed each SNP in the panel contributed equally to sICAM1 levels, and the GRA was calculated by summing up values for each SNP. The weighted GRS was calculated by multiplying the estimated beta-coefficient of each SNP by the number of corresponding risk alleles (0, 1, or 2). The values of CRP, SAA, sICAM1, sVCAM1, sE-selectin, sP-selectin, MMP-1, MMP-2, MMP-9, MCP-1, and sTNFRII levels were logarithmically transformed prior to statistical analyses to adhere to a normality assumption. P < 0.05, obtained using two-sided tests, was considered statistically significant. In addition, stepwise linear regression analysis was performed to determine independent predictors of sICAM1 levels. Associations between genotype score and plasma levels of sICAM1 was calculated using linear regression. Deviation from the Hardy–Weinberg equilibrium (HWE) and the linkage disequilibrium between polymorphisms were investigated using Golden Helix SVS Win32 7.3.1 (Golden Helix).

## Results

### Subject characteristics

We excluded carriers of the rs5491-T allele prior to all statistical analyses. Table 1 summarizes the demographic features, clinical profile, and values of a panel of inflammation markers of the study participants stratified by sex. The male subjects had higher BMI and levels of sE-selectin, sP-selectin, MMP-9, MCP-1, and sTNFRII, and a significant portion of them were current smokers (*P* < 0.05). In contrast, female subjects were slightly older and had significantly higher MMP-2 levels (*P* < 0.05).

### Associations between sICAM1 levels and cardiovascular risk factors

Unadjusted and age, sex, BMI, and smoking status-adjusted correlation coefficients between sICAM1 levels and measured cardiovascular risk factors are shown in Table 3. We found significant and positive correlations between sICAM1 levels and levels of CRP, sE-selectin, sP-selectin, sVCAM1, sTNFRII, MMP-9, and fasting serum insulin, and negative correlations between sICAM1 levels and HDL-cholesterol and adiponectin levels (*P* < 0.01). A positive correlation between sICAM1 levels and the HOMA-IR index was also found (*P* < 0.01). Adjustment for age, sex, BMI, and smoking status did not substantially alter these correlations.

**Table 3:** Association between circulatingsICAM1 levels and cardiovascular risk factors in the study subpopulation with age ≥ 18 years and without the ICAM1 rs5491-T carriers.

Clinical and biochemical parameters		Unadjusted		Adjusted for age, sex, BMI, and smoking status	
		r	*P* value	r	*P* value
Demographics	Age	0.090	0.036	0.114	0.008
	BMI	0.106	0.013	0.085	0.048
	Waist circumference	0.098	0.022	-0.012	0.776
	Weight-hip ratio	0.073	0.087	-0.007	0.877
Blood Pressure*	Systolic BP	0.070	0.104	0.007	0.878
	Diastolic BP	0.072	0.091	0.028	0.518
	Mean BP	0.076	0.075	0.020	0.644
Glucose metabolism**	Fasting plasma glucose	0.050	0.070	0.026	0.545
	Fasting serum insulin	0.167	8.82×10^−5^	0.134	0.002
	HOMA-IR index	0.162	1.36 ×10^−4^	0.132	0.002
Lipid profiles^#^	Total cholesterol	0.027	0.533	-0.005	0.899
	LDL-cholesterol	0.015	0.730	-0.0004	0.993
	HDL-cholesterol	-0.183	1.68×10^−5^	-0.149	0.001
	Triglyceride	0.172	5.17×10^−5^	0.100	0.020
Inflammation marker	CRP	0.212	5.42×10^−7^	0.169	7.36×10^−5^
	Fibrinogen	0.110	0.010	0.081	0.059
	sE-selectin	0.367	6.86×10^−19^	0.350	4.28×10^−17^
	sP-selectin	0.165	1.02×10^−4^	0.148	0.001
	sVCAM1	0.377	5.90×10^−20^	0.384	1.22×10^−20^
	sTNFRII	0.179	2.59×10^−5^	0.153	3.32×10^−4^
	MCP-1	-0.19	0.662	-0.030	0.487
	MMP-1	0.029	0.502	0.031	0.477
	MMP-2	0.002	0.968	0.017	0.685
	MMP-9	0.154	3.18×10^−4^	0.140	0.001
	SAA	0.074	0.086	0.051	0.236
Adipokines	Leptin	0.050	0.246	0.039	0.359
	Resistin	0.026	0.542	0.019	0.666
	Lipocalin2	0.012	0.782	-0.009	0.840
	Adiponectin	-0.146	0.001	-0.120	0.005

Abbreviations as Table 1. BP, blood pressure; HDL, high-density lipoprotein; LDL, low-density lipoprotein; HOMA-IR, homeostasis model assessment of insulin resistance; eGFR, estimated glomerular filtration rate; 8-OHdG: 8-hydroxy-2-deoxyguanosine.

BP levels and lipid variables were analyzed with the exclusion of subjects using antihypertensive drugs and/or lipid-lowering agents. Fasting plasma glucose and insulin and HOMA-IR index were analyzed with the exclusion of anti-diabetic medications. CRP level was calculated with the exclusion of subjects with CRP levels above 10 mg/L. Microalbuminuria/creatinine was calculated with the exclusion of subjects with Microalbuminuria/creatinine>300.

### Associations between sICAM1 levels and various SNPs of the candidate genes

We genotyped a panel of SNPs in *ICAM1* and several NF-κB pathway genes involved in chronic inflammation, and tested the associations between these SNPs and sICAM1 levels (Table 2). These SNPs included rs5496, rs5498, and rs281432 of *ICAM1*, rs28720239 of *NFKB1*, rs2230926 of *TNFAIP3*, rs12142086 of *IKBKE*, and rs16972959 of *PRKCB*. Our data showed that sICAM1 levels were associated with rs5498 of *ICAM1* (*P* = 0.002), thus corroborating the previous findings regarding the self-regulation of ICAM1 protein expression. However, we failed to detect any significant association between SNPs in the above NF-κB pathway genes and sICAM1 levels. We then tested the associations between sICAM1 levels and additional four SNPs: rs2271751, rs7894407, and rs7089271 of *PDCD11* (Program Cell Death 11), and rs1635 of *NKAPL* (NF-κB activating protein-like). PDCD11 is a NFKB1-binding protein responsible for the biogenesis of rRNA, and NKAPL is a paralogue of the NF-κB activating protein NKAP. Our data revealed that only rs1635 of *NKAPL* was significantly associated with sICAM1 levels (*P =* 0.004). The differences remained significant even after the use of stringent Bonferroni correction for multiple tests (*P* = 0.020 and *P* = 0.040, for rs5498 and rs1635, respectively).

We also searched in the GTEx database for SNPs that had cis-eQTL effect on ICAM1 (S2 Table). The SNP rs192560669 was genotyped in 100 subjects but the MAF was zero. Likewise, based on the CHB genotype data from HapMap, the MAF of rs192560669 was also zero. We then selected rs281438 which has a MAF ≥ 0.10 in the Asian populations, and strong linkage with both rs281436 and rs281437. Our association analysis showed that after adjusting for age, sex, BMI, and smoking status, rs281438 remained modestly associated with sICAM1 (*P* = 0.043) in a recessive model (data not shown). Since rs281438 had strong linkage with rs281437 according to the GTEx database, our result thus tentatively lent support to a previous finding by Bielinski et al. on the association between rs281437 and sICAM1 levels [7]. The association between rs281438 and sICAM1 levels became insignificant after Bonferroni correction, however.

### Stepwise regression and risk allele analyses of sICAM1 levels

**Fig 1:**
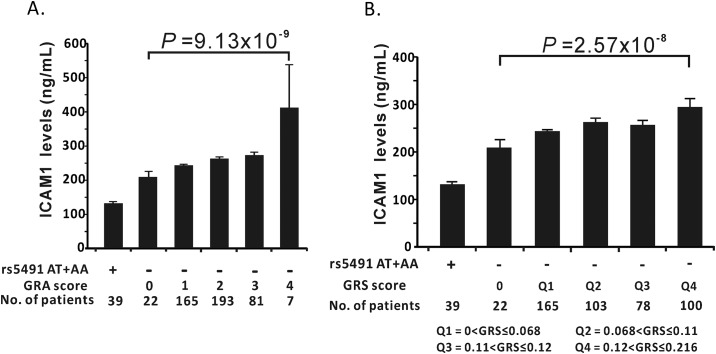
Levels of sICAM1 among non-rs5491-T allele carriers corresponding to different number of risk alleles in rs5498 of *ICAM1*, rs1635 of *NKAPL*, rs12051272 of *CDH13* and non-O of *ABO*. The associated results were presented as GRA model (A) and GRS model (B). **(**A) The sICAM1 levels (mean ± SD) of subjects carrying various number of risk alleles were as follows: no risk allele (4.7% of the population): 207.8 ± 80.9 mg/dL; one risk allele (35.3% of the population): 219.5 ± 69.3 mg/dL; two risk alleles (41.2% of the population): 261.8 ± 114.1 mg/dL; three risk alleles (17.3% of the population): 273.9 ± 130.0 mg/dL; four risk alleles (1.5% of the population): 417.9 ± 332.8 mg/dL; *P* = 9.13 × 10^−9^ after adjusting for age, sex, BMI, and smoking status. (B) The sICAM1 levels (mean ± SD) were calculated for each quartile of the GRS as follows: GRS = 0 (4.7% of the population): 207.8 ± 80.9 mg/dL; first quartile (35.3% of the population): 219.5 ± 69.3 mg/dL; second quartile (22.0% of the population): 259.6 ± 103.9 mg/dL; third quartile (16.7% of the population): 256.4 ± 99.7mg/dL; fourth quartile (21.4% of the population): 288.9 ± 166.8 mg/dL; *P* = 2.57 × 10^−8^ after adjusting for age, sex, BMI, and smoking status.

We next studied the effects of various risk variables on sICAM1 levels using stepwise linear regression adjusted for sex, age, BMI, smoking status, and the *ABO* histo-blood group genotypes (non-O and O) that were previously shown to affect sICAM1 levels (Table 4). These risk variables included the smoking status, age, the *ABO* histo-blood group genotypes, rs12051272 of *CDH13*, rs5498 of *ICAM1*, and rs1635 of *NKAPL*. Our data showed that rs5498 of *ICAM1*, rs1635 of *NKAPL*, the *ABO* genotypes, and rs12051272 of *CDH13* all independently associated with sICAM1 levels (*P* = 0.001, 0.001, 0.006, and 0.031, respectively). We then quantified the effects of a combination of risk alleles of four SNPs on sICAM1 levels. The SNPs used in this analysis were rs5498 of *ICAM1*, rs1635 of *NKAPL*, rs12051272 of *CDH13*, and non-O of ABO genotype. Our data revealed that sICAM1 levels increased significantly with increasing number of risk alleles in both the GRA and the GRS models (Fig 1, *P* = 9.13 × 10^−9^ for GRA and *P* = 2.57 × 10^−8^ for GRS, respectively) (Fig 1).

**Table 4:** sICAM1 levels: stepwise linear regression analysis, including genotypes.

Variables	Beta	R^2^	*P* value
smoking	0.072	0.024	< 0.001
*KAPL* rs1635-CC genotype	0.068	0.046	0.001
*ICAM1* rs5498-GG genotype	0.052	0.064	0.001
age	0.002	0.082	0.002
*ABO* genotypes-non-O	0.042	0.098	0.006
*CDH13*rs12051272-TT genotype	0.054	0.108	0.031

P values were adjusted for sex, age, BMI, smoking status.

## Discussion

This study analyzed the association between variants of *ICAM1* and selected NF-κB pathway genes and circulating sICAM1 levels in a Taiwanese population. Our data revealed that two SNPs, rs5498 of *ICAM1* and rs1635 of *NKAPL*, were significantly associated with sICAM1 levels. We found while sICAM1 levels were positively associated with age, HOMA-IR index, and circulating levels of CRP, sE-selectin, sP-selectin, sVCAM1, sTNFRII, MMP-9, and fasting serum insulin, they were negatively associated with levels of HDL-cholesterol and adiponectin. Using multivariate analyses, our data showed that rs5498 of *ICAM1*, rs1635 of *NKAPL*, the *ABO* genotypes, and rs12051272 of *CDH13* were all independently associated with sICAM1 levels. Finally, from our analysis of the effect of a combination of risk alleles of four SNPs on sICAM1 levels, we showed that sICAM1 levels increased significantly with increasing number of risk alleles. These results will certainly enrich our understanding of ICAM1’s involvement in cardiovascular atherosclerosis.

The SNP rs1635 of the *NKAPL* gene encodes a threonine-to-glutamine substitution at amino acid 152 in exon 1 of *NKAPL*, and was previously shown to associate with schizophrenia in separate Han Chinese populations [31–33]. Although *NKAPL* was found to be expressed in the brain in postnatal day 0 mice [33], it was still elusive regarding how a structural change in the NKAPL protein caused malfunction in the nervous system in humans, and no indication of its involvement in inflammation was provided. The rs1635 mutation might alter protein folding, protein-protein interaction, or protein expression and subcellular localization and contribute to the phenotype (i.e., sICAM1 levels) we observed in this study. Indeed, when we carried out *in silico* analysis using MutationAssesor (http://mutationassesor.org) [34], we did find medium functional impact of the T152N mutation. Moreover, according to the HapMap database (release #24, CHB), rs1635 is in linkage disequilibrium (LD) with another *NKAPL* SNP rs12000 which is a missense mutation linked to Schizophrenia [31, 32]. According to these previous studies and our own *in silico* finding, the combination of these two missense mutations can possibly alter NKAPL protein structure synergistically, which may partially explain the defects in neuronal wiring and central nervous system development seen in the Schizophrenia patients. Interestingly, the T152N mutation of rs11635 and the Y96C mutation of rs12000 potentially disrupt an AGC/GRK/BARK/BARK1 and an Abl phosphorylation site (score = 30.786, cutoff = 7.635 and score = 6.055, cutoff = 2.697, respectively) based on online predictions (http://gps.biocuckoo.org). These results suggested that the rs1635 mutation might possibly generate gain-, switch-, or loss-of-function of a domain of the NKAPL protein.

Recently, a knock-out mice study has found that NKAPL, like its paralogue and ancestral form NKAP from which it was derived, functions as a transcriptional corepressor of the Notch signaling pathway [35]. Unlike NKAP, it was found to be specifically expressed in the testes and possibly required for the self-renewal–differentiation balance of the spermatogonial stem cells. Since the authors did not provide a detailed temporal expression profile of *NKAPL* using either qRT-PCR or whole-mount *in situ* hybridization, it might be possible that it is expressed in tissues other than the testes in adulthood, and its role in the cardiovascular system may be overlooked. The discovery of its involvement in Notch signal transduction is particularly intriguing because the Notch–NF-κB signaling axis has been clearly demonstrated in the inflammatory and myeloproliferating processes in the bone marrow stromal environment [36]. It is reasonable to hypothesize that the NKAPL protein is kept at a low level in the endothelial cells of the cardiovascular system to restrain Notch signaling, and its dysregulation activates the NF-κB pathway which ultimately leads to cleavage and release of ICAM1 into the blood stream. Future research is needed to study whether NKAPL participates in this scenario, whether it represses the transcription of any NF-κB signaling activator, and whether its gain- or loss-of-function leads to NF-κB-dependent inflammatory responses in the cardiovascular environment (e.g. cytokine and sICAM1 production).

In addition to its association with sICAM1 levels, the *ICAM1* SNP rs5498 has been shown to associate with a plethora of diseases in Asians including diabetic microvascular complications [37], proliferative diabetic retinopathy [38], oral carcinogenesis [39], migraine [40], and coronary atherosclerosis [41]. Because rs5498 encodes a lysine-to-glutamic acid mutation at position 469 (K469E) which alters the electrostatic property of the binding site for ICAM1’s ligand LFA-1 [42], it is likely that changes in ICAM1’s affinity to LFA-1 contribute to the inflammation-related diseases. However, a recent study using atomic force microscopy did not find any difference in single-molecule adhesion ability between the K469 and the E469 isoforms [43]. Instead, the ICAM1 isoform containing G241 and E469 was expressed at a significantly higher level than that of the other isoforms. The G241R mutation of ICAM1, encoded by the SNP rs1799969, is located in the binding domain for the macrophage-1 antigen [42]. Therefore, these data suggest that the susceptibility of rs5498 to diseases possibly lies in differential ICAM1 protein expression rather than differential LFA-1 binding, and the differential protein expression is dependent on specific residues at both the LFA-1 and MAC-1 binding sites. Furthermore, since production of sICAM1 has been attributed to a consensus MMP-9 cleavage site (the P-X-X-Hy-T motif where X is any and Hy is a hydrophobic residue) at position 404–408 N-terminal to the transmembrane domain of ICAM1 [44], the rs5498 mutation possibly does not alter the susceptibility of ICAM1 to proteolytic cleavage. Based on the above evidence, the difference in circulating sICAM1 concentration seen in rs5498 carriers in our study is thus likely a consequence of a difference in the amount of ICAM1 expressed in the endothelial cells and the other tissues.

In recent years, a number of approaches have been proposed to address the inadequacy of traditional association studies, which are often limited in their scope and ability to detect genetic variants that may be potentially relevant to cardiovascular diseases. One type of approach, pathway analysis and related techniques, has become an essential tool for discovering key genetic components of cardiovascular diseases from large-scale GWAS data. For example, statistical techniques like gene set analysis or gene set enrichment analysis have been applied to GWAS datasets to uncover significant KEGG (Kyoto Encyclopedia of Genes and Genomes) pathways for dilated cardiomyopathy and calcific aortic valve disease [45, 46]. Another approach, which is slightly different but nonetheless cogent, uses previously-published data of cardiovascular disease-related genes to query the SNP database. The selected SNPs are then either used to build gene-centric chips or scored directly for associations [47, 48]. In this study, we took a similar candidate SNP approach and discovered a new player in the NF-κB pathway previously unknown to affect circulating sICAM1 concentration. Our results therefore suggest that pathway-informed candidate gene approach can be a handy tool to complement traditional association studies, and it vigorously accelerates the discovery of disease-relevant genes.

In this study we also demonstrated the use of both the unweighted and weighted genetic risk score (GRA and GRS, respectively) of selected SNP alleles in directly predicting the sICAM1 levels. Previously, the GRS has been extensively used to discover the underlying genetic architecture of disease traits. For instance, by informed selection of relevant SNPs according to the results of GWAS and other association studies, it has been used to predict the risks of gestational diabetes mellitus, newborn adiposity, and hypertension in different populations [49, 50]. Different GRSs were also constructed to show associations with various lipid traits and levels of CRP [51, 52]. Our study provided another example of the application of the GRS in predicting biomarker levels which might in turn point to the severity of inflammatory diseases. Further studies will be required to determine if the GRS can also be used in prognosis of long-term cardiovascular adverse events in our sample population.

## Conclusion

Our data suggested that most of the NF-κB pathway gene variants analyzed in our hands were not associated with sICAM1 levels. Nevertheless, with the finding of the association of *NKAPL* rs1635 with sICAM1 levels, it might be possible to uncover more variants of the NF-κB pathway genes that influence sICAM1 levels in Taiwanese in the future.

### Limitations

One limitation of this study is the relatively low number of subjects genotyped; replication of the current results in a second cohort would support the strength of the study. Independent association studies with larger sample size and functional data are required to confirm our results before any definitive conclusions can be drawn. Another limitation is the study's cross-sectional design, which means that the results may be used to draw only limited inference regarding the relationship between exposure and outcome. Our study design and SNP selection criteria may not be comprehensive. While some SNPs were not included into this study, our conclusion may be biased towards emphasizing the importance of certain members of the NF-κB pathway.

## Supporting Information

S1 Materials and Methods Supplementary Materials and Methods. (DOCX)

S1 Table Primer sequences used in genotyping. (DOCX)

S2 Table The GTEx database for SNPs that had cis-eQTL effect on ICAM1. (DOCX)

## Abbreviations

BMI: body mass index
BP: blood pressure
CDH13: glycosylphosphatidylinositol-anchored cadherin 13
CRP: C-reactive protein
GRA: genetic risk allele
GRS: genetic risk score
GWAS: genome-wide association studies
HDL-C: high-density lipoprotein- cholesterol
LDL-C: low-density lipoprotein-cholesterol
LFA-1: lymphocyte function-associated antigen-1
MCP-1: Monocyte chemotactic protein-1
MMP1: matrix metalloproteinase 1
MMP2: matrix metalloproteinase 2
MMP9: matrix metalloproteinase 9
NF-κB: nuclear factor-κB
NKAPL: NF-κB activating protein-like
PDCD11: Program Cell Death 11
SAA: serum amyloid A
sE-selectin: soluble E-selectin
sICAM1: soluble intercellular adhesive molecule 1
SLE: systemic lupus erythematosus
SNP: single nucleotide polymorphism
sP-selectin: soluble P-selectin
sTNFRII: soluble tumor necrosis factor-alpha receptor 2
sVCAM1: soluble vascular cell adhesive molecule 1
TNFAIP3: tumor necrosis factor-alpha-induced protein 3
