# Supplementary material for: Association between NF-κB Pathway Gene Variants and sICAM1 Levels in Taiwanese
Source: PLoS One. 2017 Jan 17;12(1):e0169516. doi: 10.1371/journal.pone.0169516 (PMC5240939; doi:10.1371/journal.pone.0169516)
Supplement: S1 Materials and Methods — (DOCX) [file pone.0169516.s001.docx]

**Supplementary Materials and Methods**

All in-house kits showed good correlation when compared with commercially available ELISA kits [1-6].

1. Chang PY, Wu TL, Tsao KC, et al. Microplate ELISAs for soluble VCAM-1 and ICAM-1. Ann Clin Lab Sci 2005; 35:312-7.
2. Tsao KC, Chang PY, Li CC, Wu TL, Sun CF, Wu JT. Development of a microplate ELISA for circulating E-selectin: assay characterization, comparison with a commercial kit, wand establishment of normal reference values. J Clin Lab Anal 2003; 17:97-101.
3. Wu TL, I Chen Tsai, Chang PY, et al. Establishment of an in-house ELISA and the reference range for serum amyloid A (SAA): complementarity between SAA and C-reactive protein as markers of inflammation. Clin Chim Acta 2007; 376:72-6.
4. Wu TL, Tsao KC, Chang CP, Li CN, Sun CF, Wu JT. Development of ELISA on microplate for serum C-reactive protein and establishment of age-dependent normal reference range. Clin Chim Acta 2002; 322:163-8.
5. Wu TL, Chang PY, Li CC, et al. Microplate ELISA for urine microalbumin: reference values and results in patients with type2 diabetes and cardiovascular disease. Ann Clin Lab Sci 2005; 35:149-54.
6. Chiou CC, Chang PY, Chan EC, et al. Urinary 8-hydroxydeoxyguanosine nd its analogs as DNA marker of oxidative stress: development of an ELISA and measurement in both bladder and prostate cancers. Clin Chim Acta 2003; 334:87-94.
