# Supplementary material for: Association between NF-κB Pathway Gene Variants and sICAM1 Levels in Taiwanese
Source: PLoS One. 2017 Jan 17;12(1):e0169516. doi: 10.1371/journal.pone.0169516 (PMC5240939; doi:10.1371/journal.pone.0169516)
Supplement: S1 Table — (DOCX) [file pone.0169516.s002.docx]

**Supplementary Table 1**. Primer sequences used in genotyping

| Gene | SNP | HWE | Primer sequence | Restriction  enzyme | PCR product |
| --- | --- | --- | --- | --- | --- |
| *ICAM1* | rs5491 | 0.828 | TaqMan SNP Genotyping Assays |  |  |
| *ICAM1* | rs5496 | - | TaqMan SNP Genotyping Assays |  |  |
| *ICAM1* | rs5498 | 0.851 | F: 5’-AGGATGGCACTTTCCCACT-3’ | BstUI | 140 bp |
|  |  |  | R: 5’-GGCTCACTCACAGAGCACAT-3’ |  |  |
| *ICAM1* | rs281432 | 0.961 | F: 5’-GCCCAGGAATTTGAGGTTACAG-3’ | Dde I | 368 bp |
| *ICAM4* | rs281438 | 0.912 | TaqMan SNP Genotyping Assays |  |  |
|  |  |  | R: 5’-GCAGTCCTTTACCAAATCCTGG-3’ |  |  |
| *NFκB1* | rs28720239 | 0.998 | F: 5’-CATGCCGACCCTCCAGCTGCTC-3’ | Pfim I | 164 bp |
|  |  |  | R: 5’-AGGGCTGGAGCCGGTAGGGAAG-3’ |  |  |
| *PDCD11* | rs2271751 | 0.771 | TaqMan SNP Genotyping Assays |  |  |
| *TNFAIP3* | rs2230926 | 1 | TaqMan SNP Genotyping Assays |  |  |
| *NKAPL* | rs1635 | 0.992 | TaqMan SNP Genotyping Assays |  |  |
| *IKBKE* | rs12142086 | 0.868 | TaqMan SNP Genotyping Assays |  |  |
| *PRKCB* | rs16972959 | 0.473 | TaqMan SNP Genotyping Assays |  |  |

HWE: Hardy-Weinberg equilibrium.
