# Supplementary material for: Association between NF-κB Pathway Gene Variants and sICAM1 Levels in Taiwanese
Source: PLoS One. 2017 Jan 17;12(1):e0169516. doi: 10.1371/journal.pone.0169516 (PMC5240939; doi:10.1371/journal.pone.0169516)
Supplement: S2 Table — (DOCX) [file pone.0169516.s003.docx]

**Supplementary Table 2.** The GTEx database for SNPs that had cis-eQTL effect on ICAM1

| SNP | Gene | MAF(CHB) | P-Value | TaqMan probe |
| --- | --- | --- | --- | --- |
| rs192560669 | ICAM5 | 0 | 5.90E-08 | X |
| rs3093029 | ICAM4 | 0.0109 | 2.20E-07 | V |
| rs75231016 | FDX1L | 0.0218 | 0.0000011 | V |
| rs73510898 | ZGLP1 | 0.0228 | 0.0000012 | X |
| rs281436 | ICAM1 | 0.126 | 0.0000024 | X |
| rs140735577 | ZGLP1 | 0 | 0.0000025 | X |
| rs281437 | ICAM1 | 0.081 | 0.0000025 | V |
| rs5030370 | ICAM1 | 0.126 | 0.0000074 | X |
| rs281430 | ICAM1 | 0.133 | 0.000014 | X |
| rs5030390 | ICAM1 | 0 | 0.000014 | X |
| rs281438 | ICAM4 | 0.122 | 0.000015 | V |
| rs281431 | ICAM1 | 0.083 | 0.000015 | X |
| rs281428 | ICAM1 | 0.111 | 0.000019 | X |
| rs5030352 | ICAM1 | 0.133 | 0.000033 | X |
| rs28382805 | CDC37 | 0.326 | 0.000045 | X |

MAF: minor allele frequency; CHB: Han Chinese in Bejing
